# Biocontrol potential of *Bacillus subtilis* CTXW 7-6-2 against kiwifruit soft rot pathogens revealed by whole-genome sequencing and biochemical characterisation

**DOI:** 10.3389/fmicb.2022.1069109

**Published:** 2022-12-01

**Authors:** Tingting Chen, Zhuzhu Zhang, Wenzhi Li, Jia Chen, Xuetang Chen, Bince Wang, Jiling Ma, Yunyun Dai, Haixia Ding, Weizhen Wang, Youhua Long

**Affiliations:** ^1^Research Center for Engineering Technology of Kiwifruit, College of Agriculture, Institute of Crop Protection, Guizhou University, Guiyang, China; ^2^Department of Plant Pathology, Guizhou University, Guiyang, China; ^3^Teaching Experimental Factory, Guizhou University, Guiyang, China

**Keywords:** kiwifruit soft rot, *Bacillus subtilis*, biocontrol, pulcherrimin, antifungal

## Abstract

Soft rot causes significant economic losses in the kiwifruit industry. This study isolated strain CTXW 7-6-2 from healthy kiwifruit tissue; this was a gram-positive bacterium that produced the red pigment pulcherrimin. The phylogenetic tree based on 16S ribosomal RNA, *gyrA, rpoB*, and *purH* gene sequences identified CTXW 7-6-2 as a strain of *Bacillus subtilis*. CTXW 7-6-2 inhibited hyphal growth of pathogenic fungi that cause kiwifruit soft rot, namely, *Botryosphaeria dothidea*, *Phomopsis* sp., and *Alternaria alternata*, by 81.76, 69.80, and 32.03%, respectively. CTXW 7-6-2 caused the hyphal surface to become swollen and deformed. Volatile compounds (VOC) produced by the strain inhibited the growth of *A. alternata* and *Phomopsis* sp. by 65.74 and 54.78%, respectively. Whole-genome sequencing revealed that CTXW 7-6-2 possessed a single circular chromosome of 4,221,676 bp that contained 4,428 protein-coding genes, with a guanine and cytosine (GC) content of 43.41%. Gene functions were annotated using the National Center for Biotechnology Information (NCBI) non-redundant protein, Swiss-Prot, Kyoto Encyclopedia of Genes and Genomes, Clusters of Orthologous Groups of proteins, Gene Ontology, Pathogen–Host Interactions, Carbohydrate-Active enZYmes, and Rapid Annotations using Subsystem Technology databases, revealing non-ribosomal pathways associated with antifungal mechanisms, biofilm formation, chemotactic motility, VOC 3-hydroxy-2-butanone, cell wall-associated enzymes, and synthesis of various secondary metabolites. antiSMASH analysis predicted that CTXW 7-6-2 can produce the active substances bacillaene, bacillibactin, subtilosin A, bacilysin, and luminmide and has four gene clusters of unknown function. Quantitative real-time PCR (qRT-PCR) analysis verified that *yvmC* and *cypX*, key genes involved in the production of pulcherrimin, were highly expressed in CTXW 7-6-2. This study elucidates the mechanism by which *B. subtilis* strain CTXW 7-6-2 inhibits pathogenic fungi that cause kiwifruit soft rot, suggesting the benefit of further studying its antifungal active substances.

## Introduction

Kiwifruit is appreciated not only for its taste but also for its abundant nutrients and dietary fibre, which are beneficial to human health (He et al., 2019). Kiwifruit originated in China and is commonly grown in New Zealand, the USA, Chile, and some European countries, making it an economically important fruit with great potential for development. Owing to the industry’s rapid development, diseases have become increasingly prominent during the cultivation, harvesting, and storage of kiwifruit, including crown gall (He et al., 2021), bacterial canker (Song et al., 2019), blossom blight (Balestra et al., 2008), leaf spot disease (Chen et al., 2022a), and soft rot (Wang et al., 2021). Among them, soft rot is the most common disease during the storage and sale of kiwifruit. Soft rot outbreaks have occurred in New Zealand, China, South Korea, and other countries, causing significant economic losses and threatening the development of the global kiwifruit industry (Manning et al., 2016). Infected fruit are asymptomatic in the early storage period, gradually showing symptoms during the post-ripening period. During infection, the epidermis of the diseased site collapses and becomes soft, round or oval-shaped brown lesions appear, and the pulp turns yellow–green, resulting in rotten and inedible fruit. Several pathogenic fungi cause soft rot, including *Botryosphaeria dothidea*, *Botrytis cinerea*, *Phomopsis* sp., and *Alternaria alternata*.

Despite considerable progress in the management and control of soft rot, kiwifruit remain vulnerable to infection. Kiwifruit are usually soaked or sprayed with fungicides before harvesting, and bagging is used to reduce the spread of dangerous pathogens. However, these measures may increase chemical residue levels in the fruit or promote fungal resistance. Therefore, researchers are urgently seeking natural antifungal active substances as alternatives to traditional fungicides. For example, plant-derived methyl jasmonate was found to effectively inhibit the growth of *B. dothidea* hyphae to protect against kiwifruit soft rot (Pan et al., 2019). Natamycin, a natural antimicrobial, inhibited *B. dothidea* growth by causing the vacuolation and plasmolysis of hyphal cells, thereby reducing kiwifruit soft rot (Pan et al., 2022). In addition, Meng et al. (2022) reported that lemon essential oil-based nanoemulsions inhibited the growth of *Phomopsis* sp. to reduce postharvest decay of kiwifruit. In terms of microbial control, a culture filtrate of *Bacillus amyloliquefaciens* effectively inhibited *B. dothidea* growth and maintained kiwifruit quality during storage (Pang et al., 2020). Further, *Bacillus subtilis* was shown to antagonise pathogenic bacteria that cause kiwifruit soft rot (Kim et al., 2015) and promote the root growth of kiwifruit stem cuttings (Erturk et al., 2010). However, the mechanism by which *B. subtilis* counteracts soft rot-causing pathogens in kiwifruit remains unclear.

*Bacillus subtilis* can produce biologically active secondary metabolites and is widely used in medicine and agriculture (Caulier et al., 2019; Su et al., 2020), in the crucial role of controlling plant diseases. *Bacillus* spp. produce various antibacterial substances that can inhibit the normal growth of plant pathogens, including bacteriocins, lanthiopeptin, and polyketides (Sumi et al., 2015; Olishevska et al., 2019), and lipopeptide metabolites that can regulate plant hormones and inhibit pathogen proliferation (Cochrane and Vederas, 2016). Moreover, *Bacillus* spp. can form biofilms (Zeriouh et al., 2014), facilitating their survival and reproduction on plant surfaces and reducing pathogen abundance (Seneviratne et al., 2010). In addition, *Bacillus* spp. produce volatile compounds (VOC) that can control *Fusarium oxysporum* sp. spore germination (Wu et al., 2020), change the cell structure of *Clavibacter michiganensis* ssp. to prevent bacterial ring rot in potatoes (Rajer et al., 2017).

Researchers have applied omics techniques to determine the genetic components related to plant growth promotion, biosynthesis of secondary metabolites, and habitat adaptation of beneficial microorganisms (Zeng et al., 2018). The goal of the present study was to isolate a bacterial strain from healthy kiwifruit with biocontrol potential against soft rot disease and elucidate the underlying mechanism using phenotypic, phylogenetic, and comparative genomics, gene and protein sequence annotation analysis, and metabolic cluster prediction.

## Materials and methods

### Microorganisms and growth conditions

The tested pathogens were obtained from the Research Center for Engineering Technology of Kiwifruit at Guizhou University, including *B. dothidea* (OM802621), *Phomopsis* sp. (OP740383), *Rhizoctonia solani* (OK287282), *Monilinia fructicola* (MW281764), *Fusarium fujikuroi* (OL774567), *A. alternata* (MG189596.1), *Colletotrichum spaethianum* (OL347722), *Pseudomonas syringae* pv. *actinidiae* (NZ_CP032631.1), and *Pantoea agglomerans* (ON955285.1). The pathogenic fungi were cultured in potato dextrose agar (PDA: 250 g potato, 20 g glucose, and 15 g agar/L) for 5 days at 28°C in the dark. The pathogenic bacteria were cultured in beef extract peptone agar (NA: 5 g beef extract, 10 g peptone, 5 g NaCl, and 15 g agar/L) for 2 days at 28°C in the dark.

### Isolation and screening of antagonistic strains

The kiwifruit were picked in an orchard in Xiuwen County, Guizhou Province (26°45′N, 106°65′E) in October 2021, packed in sterile sampling bags, and stored at 4°C. The kiwifruit surface was washed with sterile water to remove impurities, followed by soaking in 75% ethanol for 60 s, and subsequent washed with sterile water to remove the residual alcohol. Whole kiwifruit were crushed and 1 ml of crushed sample was diluted 1,000 times, spread on NA plates, and incubated at 28°C for 48 h. Subsequently, single colonies with uniform colour, glossiness, and other characteristics were selected and re-inoculated on new NA plates for 48 h. The dual-culture assay for assessing growth inhibition of pathogenic fungi was performed as described by Yan et al. (2020). Preliminarily, antagonistic bacteria that inhibited the growth of pathogenic fungi were screened out. The with obvious inhibition zones were selected for further study.

### Morphological identification and housekeeping gene amplification

Antagonistic bacterial strains were preliminarily identified by referring to Buchanan and Gibbons (1984) and Dong and Cai (2001). Single bacterial colonies were picked using an inoculating needle and cultured in NA for 5 days at 28°C under 75% relative humidity. The morphological characteristics of the single colonies were observed with the naked eye and the results of Gram staining were observed using a DM500 binocular microscope (Leica Microsystems, Wetzlar, Germany). Simultaneously, single bacterial colonies were inoculated in NA and cultured at 28°C for 24 h with shaking at 180 rpm. The ultrastructure of the bacterial cells was observed using an SU8010 scanning electron microscope (SEM; Hitachi, Tokyo, Japan) with reference to the method previously reported by Hasan et al. (2014).

To further identify the taxonomy of the antagonistic bacterial strains, phylogenetic trees of the 16S ribosomal RNA, DNA gyrase subunit A (*gyrA*), RNA polymerase subunit B (*rpoB*) and phosphoribosylaminoimidazolecarboxamide formyltransferase (*purH*) genes were constructed using multilocus gene sequence analysis (Rooney et al., 2009; Kubo et al., 2011; Dhruw et al., 2020). DNA was extracted from bacterial cells using the TIANamp Bacteria DNA Kit (Tiangen Biotech Co., Ltd., Beijing, China) according to the manufacturer’s instructions. The PCR reaction was performed in a total volume of 20 μL containing 1 μL of 100 ng/μL DNA, 0.4 μL each of 10 μmol forward and reverse primers (Table 1), 10 μL of 2 × Taq Master Mix, and 8.2 μL ddH_2_O. The thermal cycling program included a denaturation step at 94°C for 30 s, an annealing step at 55 or 60°C for 30 s, and a final extension at 72°C for 10 min for 35 cycles. The obtained PCR products were separated on 1.0% w/v agarose gel at 150 V for 20 min to confirm the DNA amplification of a single product. The PCR products were sequenced by Sangon Biotech Co., Ltd., (Shanghai, China). BLAST was used to align the sequences with the National Center for Biotechnology Information (NCBI)^1^ nucleotide sequence database and the reference sequences were downloaded. Construction of multi-locus phylogenetic trees was performed using maximum likelihood, maximum parsimony, and Bayesian inference methods (Chen et al., 2022a). The sequencing data for 16S rRNA, *gyrA*, *rpoB*, and *purH* are deposited under GenBank accession numbers ON076886, ON086734, ON086735, and ON086736, respectively.

**TABLE 1:** PCR/qRT-PCR amplification primer information.

Gene	Primer pair	Primer sequence 5′–3′	Annealing temperature (°C)	Size (bp)
*16S rRNA*	27f	AGAGTTTGATCMTGGCTCAG	55	1,448
	1387r	GGGCGGWGTGTACAAGGC		
*gyrA*	gyrA-42f	CAGTCAGGAAATGCGTACGTCCTT	60	1,007
	gyrA-1066r	CAAGGTAATGCTCCAGGCATTGCT		
*rpoB*	*rpoB*-2292f	GACGTGGGATGGCTACAACT	55	1,010
	rpoB-3354r	ATTGTCGCCTTTAACGATGG		
*purH*	*purH*-70f	ACAGAGCTTGGCGTTGAAGT	55	920
	purH-1013r	GCTTCTTGGCTGAATGAAGG		
*yvmC*	*yvmC*-F	GGCATGAGGCGGCTAATCTTCTAG	60.05	24
	yvmC-R	ACAAGGGCTCTTTCTGCAAATCTCC	59.62	25
*cypX*	*cypX*-F	GGCTGATAAGACGCTGGCACTG	61.35	22
	cypX-R	CGCAATGGCTCTCGGAACTAACG	61.07	23

### *In vitro* study

The *in vitro* inhibitory activity of the antagonistic bacterial strains against pathogenic bacteria or fungi was assessed with reference to the methods described by Yan et al. (2020) and Chen et al. (2022b). Briefly, a pathogen colony with a diameter of 6 mm was placed in the centre of a PDA plate. The bacterial strains were cultured in 50 ml of NA at 28°C for 48 h with shaking at 130 rpm. The bacterial suspension was adjusted to an OD_600_ reached 1.5–2 using a NanoPhotometer-N50 instrument (Implen, Munich, Germany). Subsequently, 100 μL aliquots of bacterial suspension were applied to four disc of filter paper (diameter of 6 mm), which were symmetrically placed 25 mm away from the pathogen colony. The control group contained only pathogen colonies without bacterial strains. All treatments were repeated three times and incubated at 28°C for 5 days.

The effects of the VOCs produced by the antagonistic strains were evaluated using the double-plate pair-button method (You et al., 2021). Briefly, 150 μL of bacterial suspension (OD_600_ reached 1.5–2) was spread evenly on an NA plate, the pathogenic fungus plug (diameter = 6 mm) was placed in the centre of a PDA plate, and the two plates were sealed together. The pathogen colony diameter was measured using a ruler and hyphal growth inhibition was calculated as follows: *A* = (a1–a2)/a1 × 100, where A is the growth inhibition of the hyphae, a1 is the colony diameter of the control pathogen, and a2 is the colony diameter of the treated pathogen. The control group used 150 μL of sterile distilled water on the NA plate instead of 150 μL of bacterial suspension.

### Mycelial morphology and hyphal ultrastructure

Hyphae at the edge of the antifungal zone were selected to evaluate the antagonistic effects of the isolated bacterial strains. Morphological changes were observed using a DM500 binocular microscope with magnification multiples of 400 (Leica) and SU8010 SEM (Hitachi) (Mo et al., 2021).

### Whole-genome sequencing, assembly, and annotation

The genomic DNA of CTXW 7-6-2 was extracted using the Rapid Bacterial Genomic DNA Isolation Kit (Sangon Biotech Co., Ltd.) according to the manufacturer’s instructions. Whole-genome sequencing was performed on the Illumina HiSeq sequencing platform (San Diego, CA, USA) and established using the NEB Next^®^ Ultra™ DNA Library Prep Kit for Illumina^®^ (New England Biolabs, Ipswich, MA, USA). Trimmomatic v. 0.36 was used to filter the sequencing data to obtain high-quality data, and FastQC v. 0.11.2 was used to visually assess quality, reads, trimming, and *de novo* assembly. The next-generation sequencing data were assembled using SPAdes v. 3.5.0. GapFiller v. 1.11 was used to complement the contigs obtained by splicing, and PrInSeS-G v. 1.0.0 was used to correct editing errors and small insertions and deletions during the splicing process. The completed whole-genome sequence was submitted to NCBI under GenBank accession number JAMKCC000000000.1.

Prokka v. 1.10 was used to predict the gene elements of the assembled results, using Prodigal to predict coding genes, Aragorn to predict tRNAs, RNAmmer to predict rRNAs, and Infernal to predict miscRNAs. The Bacterial Pan Genome Analysis tool (BPGA) (Chaudhari et al., 2016) pipeline was used with default parameters, and a phylogenetic tree was constructed for the pan-/core-genome analysis of 25 representative *Bacillus* strains with sequenced genomes.

National Center for Biotechnology Information Blast+ v. 2.2.28 was used to align the protein sequences with those in the Pathogen-Host Interactions Database (PHI-base),^2^ along with those in the Clusters of Orthologous Groups of proteins (COG), Conserved Domain Database (CDD), NCBI non-redundant protein sequences (nr), and protein family (Pfam) databases. Gene Ontology (GO^3^) analysis was performed using the protein annotation results from the Swiss-Prot and TrEMBL databases and annotation information from the UniProt database. Pathway enrichment analysis was conducted using the Kyoto Encyclopedia of Genes and Genomes (KEGG) Automatic Annotation Server.^4^ HMMER3 v. 3.1b1 was used to align the gene set protein sequences with the Carbohydrate-Active enZYmes database (CAZy),^5^ thereby obtaining the corresponding carbohydrate-active enzyme annotation information (*E*-value < 10 ^––5^). The Rapid Annotations using Subsystem Technology (RAST)^6^ (Overbeek et al., 2013) approach was used to categorise the gene sets of proteins involved in specific biological processes.

### Bioactive compound discovery

The secondary metabolite synthesis gene clusters of *B. subtilis* CTXW 7-6-2 were predicted and analysed for potential bacteriostatic ability using antiSMASH v. 4.0 (Blin et al., 2017).^7^ *B. subtilis* NCIB 3610 (produces red pigment, GenBank: CP020102.1) and *B. subtilis* subsp. *subtilis* 168 (does not produce red pigment, Gen Bank: NC_000964.1) were used as control strains for analysis of secondary metabolite synthesis gene clusters.

### Bioinformatics analysis and quantitative real-time PCR of key genes involved in pulcherriminic acid production

The enzymes encoded by *yvmC* [cyclo(L-leucyl-L-leucyl) synthase] and *cypX* (pulcherriminic acid synthase) are known to be involved in the production of the red pigment pulcherriminic acid by *Bacillus* spp. (Li et al., 2017). Hence, the amino acid sequences of these two enzymes were selected from the whole gene sequence of the antagonistic bacteria. ProtParam^8^ was used to analyse the physicochemical properties of the proteins. Meanwhile, Predict Protein^9^ and SWISS-MODEL^10^ were used to predict the secondary and tertiary protein structures, respectively.

Quantitative real-time PCR (qRT-PCR) was used to determine the relative expression levels of *yvmC* and *cypX* in the antagonistic bacteria cultured at 28°C for 1 and 5 days with shaking at 180 rpm, respectively. *B. subtilis* GUMT323, which cannot produce red pigment, was used as the control strain (provided by Professor Ding Haixia of Guizhou University, CCTCC no. M 2018873). Sangon Biotech Co., Ltd., designed and synthesised the primers for *yvmC* and *cypX*, the sequences of which were submitted to their online platform (Table 1).^11^ The *rpoB* gene was used as the internal control (Jiang et al., 2021). Total RNA extraction and reverse transcription were performed using the RNAprep Pure Bacteria Kit (Tiangen Biotech) and First Strand cDNA Synthesis Kit (Tiangen Biotech), according to the manufacturer’s instructions. The cDNA was diluted to 200 ng/μL for the qPCR assay with each gene-specific primer. qRT-PCR was performed using Talent qRT-PCR SYBR Green PreMix (Sangon Biotech Co., Ltd.) on the Bio-Rad CFX96 qRT-PCR system (Bio-Rad, Hercules, CA, USA). Four biological replicates were analysed. Relative gene expression was calculated using the 2 ^–Δ^ ^Ct^ method.

### Statistical analysis

Statistical analysis was conducted using SPSS Statistics v. 13.0 software (SPSS Inc., Chicago, IL, USA). All treatments were performed using three biological replicates. Data are expressed as the mean ± standard deviation (SD). Differences between treatments were determined using one-way ANOVA and Tukey’s honest significant difference test. A *p*-value < 0.05 was considered to be statistically significant.

## Results

### Morphological identification and housekeeping gene analysis

**FIGURE 1:**
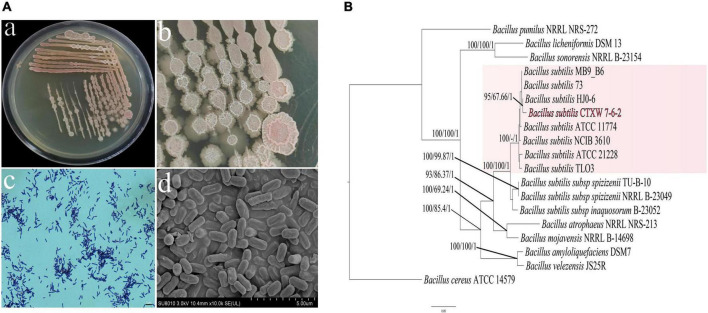
Morphological observations and analysis of housekeeping genes used to construct a phylogenetic tree. **(Aa,b)** Colony morphology in nutrient agar, **(Ac)** Gram staining. **(Ad)** Rod-shaped bacteria viewed under scanning electron microscopy. **(B)** A maximum likelihood tree was constructed using combined sequence analysis of the 16S ribosomal RNA, *gyrA, rpoB*, and *purH* genes. Bootstrap support values, maximum parsimony, and Bayesian inference posterior probabilities are given at each node based on 1000 bootstrap replicates.

Strain CTXW 7-6-2 was a rod-shaped gram-positive bacterium with dimensions of 0.62–0.75 μm × 1.5–2.0 μm (Figure 1A). After culturing for 5 days, the strain formed a single medium-sized, round, flat, and dry colony that was opaque, grey-white in colour, and later produced a light red pigment. Following PCR amplification and sequence alignment analyses of 16S rRNA, *gyrA*, *rpoB*, and *purH*, the constructed phylogenetic tree showed that strain CTXW 7-6-2 clustered with *B. subtilis*, with a self-sustaining rate of 100% maximum likelihood and 1.00 Bayesian inference. *Bacillus cereus* ATCC 14579 was shown to be an outgroup (Figure 1B).

### *In vitro* antibacterial activity of strain CTXW 7-6-2

A total of 25 bacterial strains were isolated, among which strain CTXW 7-6-2 displayed strong antagonistic effects using a double-layer double-culture assay. Table 2 lists the antagonistic effects of strain CTXW 7-6-2 against kiwifruit soft rot-causing pathogens, with growth of *Phomopsis* sp., *A. alternata*, and *B. dothidea* inhibited by 81.76 ± 3.554%, 69.80 ± 1.242%, and 32.03 ± 0.608%, respectively. Additionally, strain CTXW 7-6-2 inhibited the growth of *F. fujikuroi*, which causes leaf spot disease in kiwifruit, by 38.37 ± 2.265%. No inhibitory effect was observed against *P. syringae* pv. *actinidiae*, which causes bacterial canker in kiwifruit.

**TABLE 2:** Inhibitory effects against pathogenic fungi or bacteria.

Pathogenic fungi	Source	Colony diameter (cm)		Growth inhibition (%)
		Control	Treatment	
*Botryosphaeria dothidea*	Kiwifruit	7.26 ± 0.033	4.93 ± 0.044	32.03 ± 0.608^ef^
*Phomopsis* sp.	Kiwifruit	5.30 ± 0.126	0.97 ± 0.188	81.76 ± 3.554^b^
*Fusarium fujikuroi*	Kiwifruit	5.84 ± 0.022	3.60 ± 0.132	38.37 ± 2.265^e^
*Alternaria alternata*	Kiwifruit	5.85 ± 0.025	1.77 ± 0.073	69.80 ± 1.242^c^
*Pseudomonas syringae pv. actinidiae*	Kiwifruit	–	–	0.00 ± 0.000^g^
*Colletotrichum spaethianum*	Chinese ground orchids	5.40 ± 0.321	1.49 ± 0.036	72.38 ± 0.673^c^
*Rhizoctonia solani*	Tobacco	5.40 ± 0.153	0.36 ± 0.191	93.36 ± 3.529^a^
*Monilinia fructicola*	Plum	5.70 ± 0.043	2.46 ± 0.217	56.87 ± 3.801^d^
*Pantoea agglomerans*	Plum	–	–	0.00 ± 0.000^g^

Values represent the mean ± SD (*n* = 3). Letters indicate a significant difference at *p* < 0.05.

Strain CTXW 7-6-2 also exhibited antifungal effects against pathogenic fungi that cause diseases in tobacco, plums, and Chinese ground orchids. The most significant antagonistic effects were observed against *R. solani*, which causes target spot disease in tobacco, with the colony diameter reduced to 0.36 ± 0.191 cm and growth inhibited by 93.36 ± 3.529%. The antagonistic effects of strain CTXW 7-6-2 were also observed against *C. spaethianum*, which causes anthracnose in Chinese ground orchids, and *M. fructicola*, which causes brown rot in plums, inhibiting growth by 72.38 ± 0.673% and 56.87 ± 3.801%, respectively. Strain CTXW 7-6-2 displayed no inhibitory effect against *P. agglomerans*, which causes bacterial shot-hole disease in plums.

**FIGURE 2:**
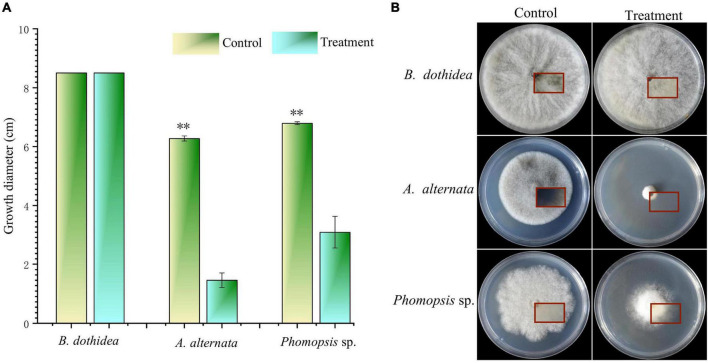
*In vitro* antagonism of the volatile compounds of strain CTXW 7-6-2 against kiwifruit soft rot-causing pathogenic fungi. Measurement of colony diameter **(A)** and colony morphology **(B)**. The red square indicated the back of the colony. *A significant difference at *p* < 0.05. **A highly significant difference at *p* < 0.01.

The VOCs of strain CTXW 7-6-2 had differing inhibitory effects against soft rot-causing pathogens in kiwifruit. The VOCs displayed no inhibitory effect against *B. dothidea* growth (Figure 2), but the colony diameter of *A. alternata* was reduced to 1.46 ± 0.25 cm, growth was inhibited by 76.76 ± 3.93%, and no melanin was produced (Figure 2B). The VOCs of strain CTXW 7-6-2 inhibited the growth of *Phomopsis* sp. by 54.48 ± 7.96% and reduced the colony diameter to 3.10 ± 0.54 cm.

### Microscopic observation of hyphae

**FIGURE 3:**
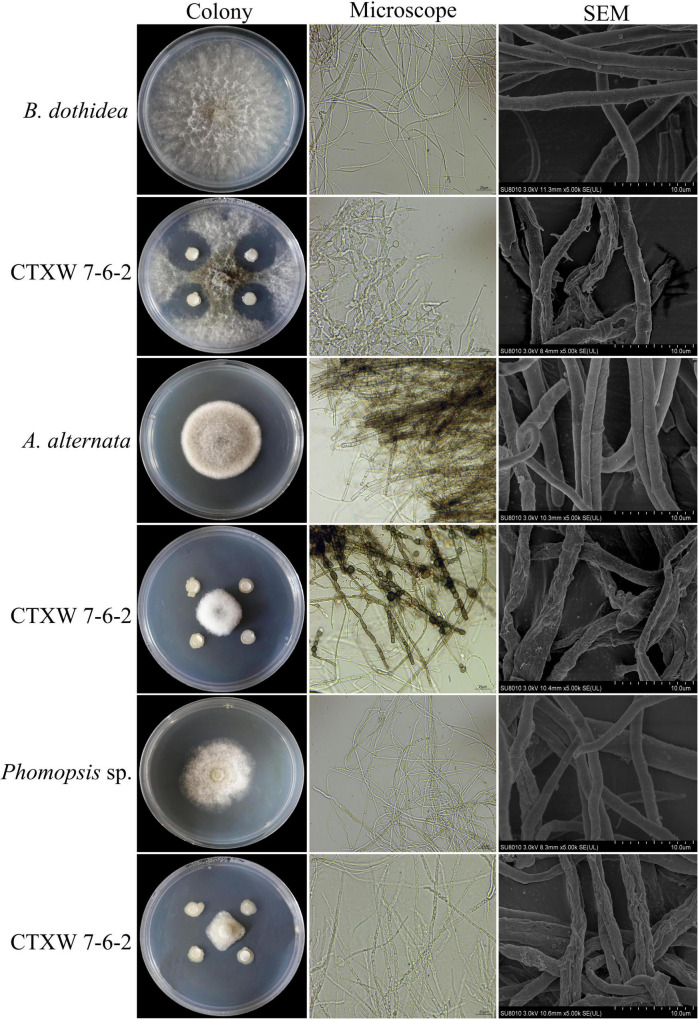
Comparison of colony and hyphal morphology of kiwifruit soft rot-causing pathogenic fungi before and after exposure to strain CTXW 7-6-2.

*Botryosphaeria dothidea*, *Phomopsis* sp., and *A. alternata* displayed obvious growth inhibition zones near CTXW 7-6-2, with slow mycelial growth. The inhibited *B. dothidea* colonies were grey-black in colour, the tops and internodes of the hyphae were swollen, and their growth was hindered. The inhibited *Phomopsis* sp. colonies had neat edges, distorted hyphae, and showed dissolution. The inhibited *A. alternata* colonies were dense and whitish in colour, and the hyphae were swollen and became bead-like at each internode (Figure 3).

The SEM images revealed that the hyphae of the three pathogenic fungi were deformed to varying degrees. The untreated hyphae of each fungal species appeared smooth and plump. In comparison, the hyphae near CTXW 7-6-2 were rough, shrunken, twisted, and otherwise deformed, indicating severe damage (Figure 3).

### Genome feature analysis and function annotation

**FIGURE 4:**
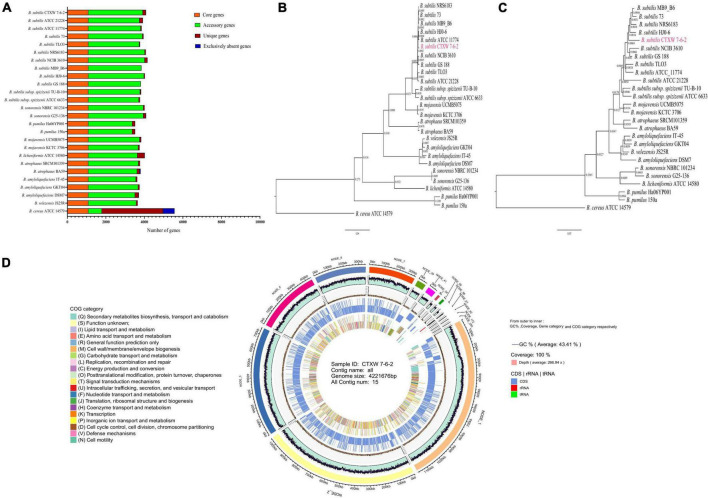
Comparison of genomes and circular genome map of strain CTXW 7-6-2. **(A)** Genome-wide proportions of core, accessory, unique, and exclusively absent genes were analysed among 26 *Bacillus* species. **(B)** Phylogenetic trees constructed based on core genomes. **(C)** Construction of phylogenetic trees based on pan-genomic analysis. **(D)** Displayed in order from outside to inside: scale bar, GC content (GC: ratio of guanine and cytosine), sequencing depth, gene element display, and clusters of orthologous group functional display.

The genomes of strain CTXW 7-6-2 and 25 representative *Bacillus* strains contained the same number of core genes and differing numbers of accessory, unique, and exclusively absent genes (Figure 4A). The phylogenetic analysis based on core and pan-genome data demonstrated that strain CTXW 7-6-2 was clearly clustered with *B. subtilis* and distinct from other *Bacillus* species (Figures 4B,C), with *B. cereus* ATCC 14579 as an outgroup. The *B. subtilis* CTXW 7-6-2 genome was finally assembled into 15 contigs with a total length of 4,221,676 bp (N50 contig size of approximately 1,046,756 bp), guanine and cytosine (GC) content of 43.41%, 4,428 protein-coding genes, 75 tRNAs, 10 rRNAs, and 45 unknown genes. A total of 3,079 annotations from the COG database were divided into 20 functional groups, mainly related to amino acid transport and metabolism, carbohydrate transport and metabolism, and transcription (Figure 4D).

**FIGURE 5:**
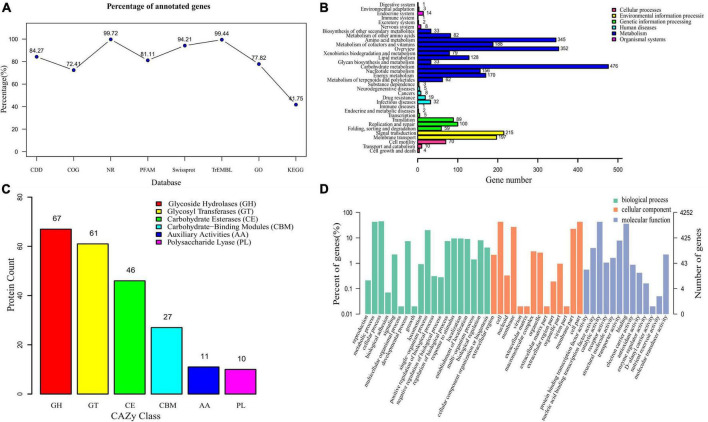
Functional analysis of gene and protein sequence annotations from *B. subtilis* CTXW 7-6-2. **(A)** Proportions of genes annotated in eight databases. **(B)** Metabolic pathways annotated using the Kyoto Encyclopedia of Genes and Genomes (KEGG) database. **(C)** Proportions of enzymes annotated using the Carbohydrate-Active enZYmes database. **(D)** Biological process, cellular component, and molecular function terms annotated using the Gene Ontology database.

The whole-genome of *B. subtilis* CTXW 7-6-2 was functionally annotated using eight gene annotation databases (Figure 5A). A total of 4,240 annotations were found in the nr database, accounting for 99.72% of all annotations. Additionally, 4,006 (94.21%), 3,583 (84.27%), 3,449 (81.11%), and 4,228 (99.44%) annotations were found in the Swiss-Prot, CDD, Pfam, and TrEMBL databases, respectively. A total of 1,775 KEGG annotations were associated with 34 metabolic pathways (Figure 5B). The genome of *B. subtilis* CTXW 7-6-2 had the highest number of genes associated with metabolism (2,104); carbohydrate, amino acid and cofactors, vitamin metabolism, and environmental information processing (412); genetic information processing (253); and cell processes (84). A total of 222 annotations were found in the CAZy database, including glycoside hydrolases (67), glycosyl transferases (61), carbohydrate esterases (46), auxiliary activities (11), carbohydrate-binding modules (27), and polysaccharide lyases (10) (Figure 5C). Glycosyl transferases, glycoside hydrolases, and carbohydrate esterase-related enzymes are involved in the synthesis of secondary metabolites *via* non-ribosomal pathways. Furthermore, *B. subtilis* CTXW 7-6-2 genome had 4 count proteins chitinase (GH18), 1 count proteins chitosanase (GH46), 13 count proteins endoglucanase (GH 5,51,16,26,11), and 8 count proteins lysozyme (GH23,73) encoding for possible antifungal CAZymes. *AlsD* (Alpha-acetolactate decarboxylase) and *alsS* (acetolactate synthase) genes encoding proteins are involved in the production of VOC 3-hydroxy-2-butanone. GO analysis revealed the main molecular functions of *B. subtilis* CTXW 7-6-2 were related to catalytic activity and binding function, while the main cellular components were related to cell and membrane parts (Figure 5D). In addition, 92 annotations were found in the PHI-base, including 10 proteins associated with loss of pathogenicity and 50 proteins associated with reduced virulence.

**FIGURE 6:**
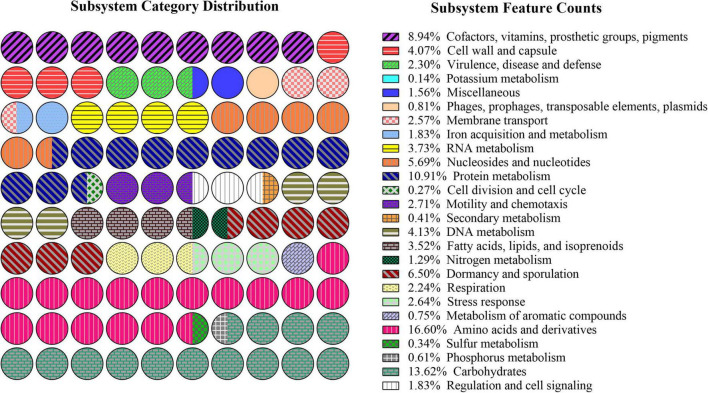
Subsystem distribution of the genome of *B. subtilis* CTXW 7-6-2 using the Rapid Annotations using Subsystem Technology (RAST) annotation server.

Rapid annotations using subsystem technology (RAST) subsystem analysis (Figure 6) revealed that the genome of *B. subtilis* CTXW 7-6-2 contained 245 genes involved in the metabolism of amino acids and their derivatives, 201 genes involved in carbohydrates, 161 genes involved in protein metabolism, 55 genes involved in RNA metabolism, and 132 genes involved in cofactors, prosthetic groups, vitamins, and pigment synthesis. The above annotation results indicated that various material and energy metabolism pathways provided the necessary nutrients and energy supply for the growth of *B. subtilis* CTXW 7-6-2. In addition, 27 genes were involved in iron acquisition and metabolism, 39 genes were involved in the stress response, 38 genes were involved in membrane transport, 40 genes were involved in motility and chemotaxis, 60 genes were involved in the cell wall and capsule, 6 genes were involved in secondary metabolism, 52 genes were involved in fatty acids, lipids, and isoprenoids, suggesting beneficial traits for potential biocontrol capabilities.

### Bioactive compound prediction

**FIGURE 7:**
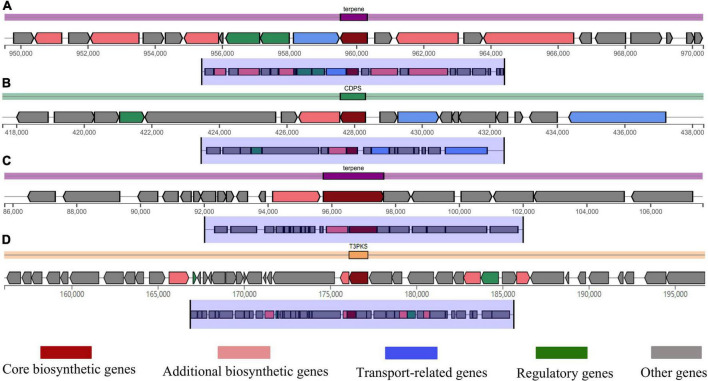
Domains of some gene clusters of *B. subtilis* CTXW 7-6-2. **(A)** Type terpene, *crtB* core gene. **(B)** Type CDPS, *yvmC* core gene. **(C)** Type T3PKS, alpha-pyrone synthesis polyketide synthase-like Pks11. **(D)** Type terpene, *sqhC* core gene.

Predicting the metabolites produced by *B. subtilis* CTXW 7-6-2 is conducive to further analysis of its antifungal compounds. The antiSMASH analysis showed that *B. subtilis* CTXW 7-6-2 had 14 secondary metabolite biosynthetic gene clusters (BGCs), including five NRPS (non-ribosomal peptide synthetase) clusters, two putative NRPS + polyketide clusters, a polyketide cluster, and a thiopeptide cluster. The bioactive compounds with a predicted similarity of 100% were identified as bacillaene, bacillibactin, subtilosin A, bacilysin, and luminmide (Table 3). *B. subtilis* CTXW 7-6-2 also had four gene clusters with unknown functions, including one CDPS (tRNA-dependent cyclodipeptide synthase) cluster, one T3PKS (Type III PKS) cluster, and two terpene clusters (Figure 7), suggesting that some *B. subtilis* CTXW 7-6-2 gene clusters may synthesise new antibacterial substances. Schedule 1 and Table 3 show that CTXW 7-6-2 shared seven and six metabolic species classes with *B. subtilis* NCIB 3610 and 168, respectively. Luminmide, plipastatin, and basiliskamide A/B were unique to *B. subtilis* CTXW 7-6-2. Both *B. subtilis* CTXW 7-6-2 and NCIB 3610 had a predicted a gene cluster of unknown function containing *yvmC* as the core gene, in contrast to *B. subtilis sub*sp. *subtilis* 168 (Schedule 1). *Bacillus* sp. that produce pulcherrimin are speculated to contain *yvmC*.

**TABLE 3:** Identification of secondary metabolite-related gene clusters in the genome of *B. subtilis* CTXW 7-6-2.

Strain	Type of secondary metabolite	Most similar known cluster	From–to (location, bp)	Similarity (%)	MIBiG BGC-ID	Core genes/Products
*B. subtilis* CTXW 7-6-2	Polyketide + NRPS	Bacillaene	253,361–358,622	100	BGC0001089	pksR + pksN + pksM + pksL + pksJ + pksG + pksF + pksE + pksC
	NRPS	Bacillibactin	82,093–129,229	100	BGC0000309	dhbF
	RiPP (Thiopeptide)	Subtilosin A	658,164–679,775	100	BGC0000602	sboA + albA
	Other	Bacilysin	682,773–724,191	100	BGC0001184	bacD
	NRPS	Luminmide	325,490–347,006	100	BGC0001128	tycC
	NRPS	Fengycin	78–39,208	86	BGC0001095	ppsC + ppsD + ppsE + lcfB_1 + yngG
	NRPS (lipopeptide)	Surfactin	198,652–264,040	82	BGC0000433	srfAC + srfAB + srfAA
	NRPS	Plipastatin	1–14,821	30	BGC0000407	ppsB + ppsA_1
	NRPS + Polyketide	Thailanstatin A	939,766–961,464	10	BGC0001114	yydH + moaA_2
	Polyketide	Basiliskamide A/Basiliskamide B	1–24,238	9	BGC0000172	fabF_3
	Terpene	–	949,524–970,327	–	–	crtB
	CDPS	–	417,587–438,333	–	–	yvmC
	T3PKS	–	156,094–197,191	–	–	Alpha-pyrone synthesis polyketide synthase-like Pks11
	Terpene	–	85,741–107,639	–	–	sqhC

–, similarity not found; NRPS, non-ribosomal peptide synthetase cluster; T3PKS, type III PKS; RiPP, ribosomally synthesised and post-translational modified peptide; CDPS, tRNA-dependent cyclodipeptide synthase; RRE-containing, RRE-element containing cluster.

### Bioinformatics analysis of *yvmC* and *cypX*

The ProtParam analysis indicated that the molecular weights of the yvmC and cypX proteins of *B. subtilis* CTXW 7-6-2 were 28,506.74 and 45,499.08 Da and contained 248 and 405 amino acids, respectively. Additionally, the yvmC and cypX proteins of *B. subtilis* CTXW 7-6-2 had 33 and 41 positively charged residues (Arg + Lys) as well as 35 and 55 negatively charged residues (Asp + Glu), respectively. Moreover, their theoretical isoelectric points were 6.57 and 5.42 and their grand average of hydropathicity index values were −0.268 and −0.128, respectively. The calculated instability index values indicated that the yvmC (46.24) and cypX (50.78) proteins of *B. subtilis* CTXW 7-6-2 were negatively charged, unstable, and hydrophilic.

**FIGURE 8:**
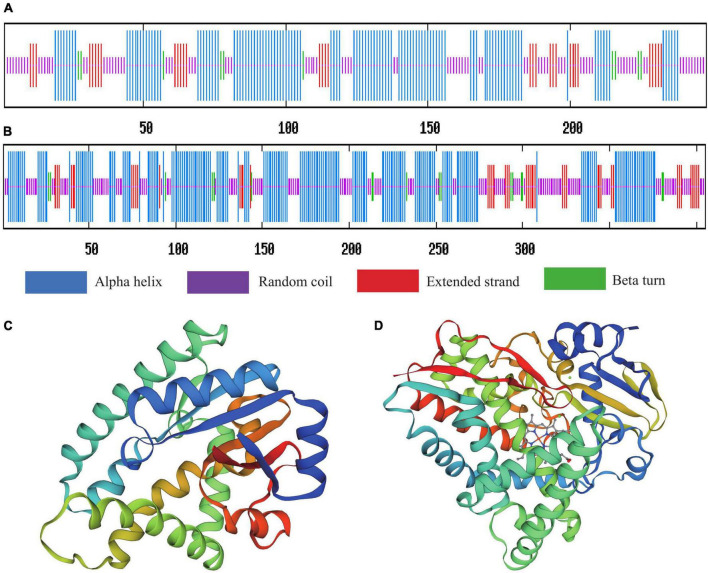
Secondary structure prediction and protein spatial conformation maps of **(A,C)** yvmC and **(B,D)** cypX of *B. subtilis* CTXW 7-6-2.

Predict Protein analysis of the secondary protein structure of yvmC showed that alpha helices, beta turns, extended strands, and random coils accounted for 48.39, 4.03, 12.90, and 34.68% of the total structure, respectively. Alpha helices, beta turns, extended strands, and random coils accounted for 50.37, 4.20, 10.86, and 34.57% of the secondary protein structure of cypX, respectively. The secondary structures of both proteins were dominated by alpha helix and random coil structures, followed by extended strands, and then beta turns (Figures 8A,B). The protein tertiary structure homology was predicted using SWISS-MODEL, as shown in Figures 8C,D.

**FIGURE 9:**
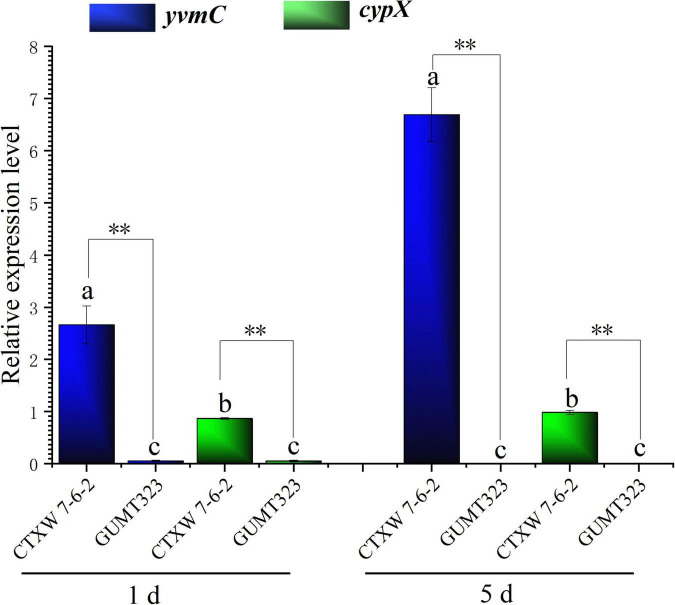
Quantitative real-time PCR analysis of *yvmC* and *cypX* gene expression in *B. subtilis* CTXW 7-6-2 and GUMT323, related to the production to pulcherriminic acid. Letters indicate significant differences between strains at *p* < 0.05, ^**^indicates a significant difference at *p* < 0.01.

The relative expression levels of *yvmC* and *cypX* in *B. subtilis* CTXW 7-6-2 increased with culture time, increasing 6.6938 ± 0.517-fold and 0.983 ± 0.042-fold on day 5 compared to that on day 1, respectively (Figure 9). *yvmC* was more highly expressed than *cypX* in *B. subtilis* CTXW 7-6-2. In contrast, *yvmC* and *cypX* were relatively less expressed in *B. subtilis* GUMT323, with expression levels of lower than 0.06-fold.

## Discussion

Soft rot threatens the yield, quality, and economic value of kiwifruit. The current study conducted a preliminary exploration of the biological control exerted by strain CTXW 7-6-2 against kiwifruit soft rot. Strain CTXW 7-6-2 was identified as a *B. subtilis* strain *via* morphological observation and combined analysis of 16S rRNA, *gyrA, rpoB*, and *purH*. Additionally, strain CTXW 7-6-2 produced a light-red pigment, which was consistent with an unknown *B. subtilis* strain isolated from coconut tissue culture (Salo and Novero, 2020). Strain CTXW 7-6-2 inhibited pathogenic fungi associated with diseases that infect kiwifruit, tobacco, plums, and Chinese ground orchids, and adversely affected the hyphae of the pathogenic fungi *B. dothidea*, *Phomopsis* sp., and *A. alternata*, which cause soft rot. Although the biologically active substances were not extracted, secondary metabolites were predicted in the current study, thus providing a theoretical basis for the extraction of active substances in the future. Additionally, the volatile substances produced by strain CTXW 7-6-2 inhibited the growth of *Phomopsis* sp. and *A. alternata*. Volatile substances produced by *Bacillus* spp. reportedly inhibit phytopathogenic fungi (Li et al., 2020; De la Cruz-López et al., 2022; Wang et al., 2022), affect soil microbial richness (Yuan et al., 2017) and promote plant growth (Ghazala et al., 2022; Rojas-Sanchez et al., 2022). Nevertheless, the mechanisms by which the strain and its volatile substances affect pathogenic fungi *in vivo* require further study.

Whole-genome sequencing and comparative genomics have become increasingly useful for exploring the molecular mechanisms and antifungal genes of bacteria as potential biocontrol agents. The whole-genome sequencing results in the current study indicated that secondary metabolites were actively involved in the antifungal mechanisms of *B. subtilis* CTXW 7-6-2. Gene and protein sequence annotation analyses predicted that many carbon, amino acid, and energy metabolism pathways were involved in iron acquisition and metabolism, motility and chemotaxis, membrane transport, VOC 3-hydroxy-2-butanone, cell wall-associated enzymes, and other related favourable traits. Moreover, glycoside hydrolases, glycosyl transferases, and carbohydrate esterase enzymes accounted for most of the identified carbohydrate-active enzymes, which are beneficial for secondary metabolite synthesis *via* non-ribosomal pathways.

The predicted secondary metabolite gene clusters of *B. subtilis* CTXW 7-6-2 revealed hitherto undetected secondary metabolite gene clusters, as well as various predicted lipopeptide antibiotics or proteins, including bacillaene, bacillibactin, subtilosin A, bacilysin, and luminmide. Bacillaene, a polyketide composed of low-molecular weight fatty acids (Chen et al., 2006), exerts antibacterial activity by inhibiting pathogenic protein synthesis. Bacillibactin is an iron chelator that can competitively bind soluble iron ions necessary for the growth and activity of pathogens (Roy and Griffith, 2017). Subtilosin A is a ribosomally synthesised and post-translationally modified peptide (RiPP) with broad-spectrum antibacterial activity that can act on and interfere with the phospholipid bilayer of various bacteria (Thennarasu et al., 2005). Bacilysin mainly disrupts the integrity of pathogenic cell membranes and damages cell wall structure (Loffler et al., 2010).

Interestingly, *yvmC* was predicted to be present in a gene cluster with unknown function in both red pigment-producing *B. subtilis* CTXW 7-6-2 and NCBI 3160, but was not predicted to be present in *B. subtilis* 168, which does not produce pulcherrimin. The formation of cyclo(L-leucyl-L-leucyl) is mediated by cyclodipeptide synthase YvmC, which utilises charged leucyl tRNAs (Sauguet et al., 2011). *cypX* encodes a cytochrome P450 implicated in the transformation of cyclo(L-leucyl-L-leucyl) into pulcherriminic acid (Max et al., 2010). Pulcherriminic acid combines with extracellular ferric ions to form the red pigment pulcherrimin in a non-enzymatic reaction. Pulcherrimin has been shown to effectively inhibit the growth of yeast and fungi (Attila et al., 2015) and affect biofilm formation (Arnaouteli et al., 2019). *yvmC* and *cypX* were highly expressed in *B. subtilis* CTXW 7-6-2 and the relative expression of *yvmC* was higher than that of *cypX*. In contrast, *yvmC* and *cypX* were expressed at lower levels in non-red pigment-producing *B. subtilis* GUMT323. Additionally, the yvmC and cypX proteins in *B. subtilis* CTXW 7-6-2 were negatively charged, unstable, hydrophilic, and rich in alpha helix and random coil structures. The study findings suggest that pulcherrimin may be partly responsible for the antagonistic effects of *B. subtilis* CTXW 7-6-2 against soft rot-causing pathogenic fungi in kiwifruit, which warrants further investigation.

## Conclusion

This study isolated *B. subtilis* strain CTXW 7-6-2 from healthy kiwifruit, which produced the red pigment pulcherrimin, inhibited the growth of soft rot-causing pathogenic fungi, and destroyed the hyphal structure of the tested fungi. *In vitro* experiments demonstrated that *B. subtilis* CTXW 7-6-2 possessed broad-spectrum antifungal activity. Whole-genome sequencing revealed the beneficial traits responsible for the antagonistic effects of *B. subtilis* CTXW 7-6-2 and predicted the production of active substances such as bacillaene, bacillibactin, subtilosin A, bacilysin, and luminmide. The key genes involved in pulcherrimin production, *yvmC* and *cypX*, were highly expressed in *B. subtilis* CTXW 7-6-2, which supports future in-depth studies of *B. subtilis*-derived pulcherrimin as an antifungal agent. The study findings may facilitate the development of natural prevention and control treatments against kiwifruit soft rot.

## Data availability statement

16S rRNA, gyrA, rpoB, and purH are deposited under GenBank accession numbers: ON076886, ON086734, ON086735, and ON086736, respectively. The completed whole-genome sequence was submitted to NCBI under GenBank accession number: JAMKCC000000000.1 (https://www.ncbi.nlm.nih.gov/nuccore/?term=Bacillus+subtilis+CTXW+7-6-2).

## Author contributions

TC and YL designed the research and wrote the manuscript. TC, ZZ, WL, XC, JC, BW, JM, and HD performed all the experiments. TC and WW analysed the data. All authors reviewed the final manuscript.

## Footnotes

1
https://www.ncbi.nlm.nih.gov/

2
http://www.phi-base.org/

3
http://www.geneontology.org

4
http://www.genome.jp/tools/kaas/

5
http://www.cazy.org/

6
https://rast.nmpdr.org/rast.cgi

7
https://antismash.secondarymetabolites.org/

8
https://web.expasy.org/protparam/

9
https://npsa-prabi.ibcp.fr/

10
https://swissmodel.expasy.org/

11
https://www.sangon.com/newPrimerDesign
